# Genomic signatures of lineage-specific structural variants in the *Pan* genus

**DOI:** 10.1093/bib/bbaf631.051

**Published:** 2025-12-12

**Authors:** Aisha Yousaf, Hua Chen

**Affiliations:** Government College University Faisalabad, Pakistan; Beijing Institute of Genomics, Chinese Academy of Sciences/China National Center for Bioinformation, Beijing, China; Beijing Institute of Genomics, Chinese Academy of Sciences/China National Center for Bioinformation, Beijing, China; Fudan University, China

## Abstract

**Background:**

Structural variants (SVs; ≥ 50 bp) represent a major source of genomic variation and are more likely to influence gene function and phenotypes than small-scale insertions/deletions or single nucleotide variants. Although, many studies have characterized SVs in humans and non-human primates, lineage-specific SVs within the *Pan* genus remain underexplored. The only species in this genus, chimpanzee (*Pan troglodytes*) and bonobo (*Pan paniscus*), exhibit lineage-specific differences for several behavioral and physiological traits.

**Aim:**

In current study, we sought to identify and characterize lineage-specific structural variants (LSSVs) in the *Pan* genus using high-quality genome assemblies, thereby providing novel insights into their lineage-specific emergence and potential functional impacts.

**Methods:**

We detected LSSVs using an assembly-comparison approach that leveraged high-quality genome assemblies of chimpanzee, bonobo, and human. We then mapped their genome-wide distributions and conducted functional enrichment analyses of genes impacted by these variants.

**Results:**

We observed substantial variation in SV abundance between the two sister species, with the chimpanzee assembly exhibiting nearly a four-fold higher SV count compared with the bonobo lineage. Genome-wide SV distribution maps revealed distinct genomic regions abundant or depleted in SVs, highlighting a marked difference in LSSV distribution across diverse genomic landscapes. Functional enrichment analysis of LSSV-impacted genes highlighted many genes related to body growth, brain function, and neurological disorders in the bonobo lineage, whereas metabolism and transcriptional regulation showed enrichment in the chimpanzee lineage. Additionally, a small subset of the SV-affected genes also corresponded to distinctive behavioral differences between the two lineages, suggesting a potential role in shaping the lineage-specific traits present within the *Pan* genus.

